# Cobertura radicular múltiple asociada a injerto de tejido conectivo con abordaje frontal modificado: reporte de caso

**DOI:** 10.21142/2523-2754-1404-2026-323

**Published:** 2026-10-01

**Authors:** María Lozano-Figueroa, Leonardo Rodríguez-Gonzales, Xiomara Alejo-Barrientos, Jonathan Meza-Mauricio

**Affiliations:** 1 Departamento de Periodontología, Facultad de Odontología, Universidad Científica del Sur. Lima, Perú. 100082251@cientifica.edu.pe 100072585@cientifica.edu.pe emezam@cientifica.edu.pe Universidad Científica del Sur Departamento de Periodontología Facultad de Odontología Universidad Científica del Sur Lima Peru 100082251@cientifica.edu.pe 100072585@cientifica.edu.pe emezam@cientifica.edu.pe; 2 Práctica privada. rodriguez.leonardoh@gmail.com Práctica privada rodriguez.leonardoh@gmail.com

**Keywords:** recesión gingival múltiple, cobertura radicular, multiple gingival recession, root coverage

## Abstract

**Objetivo:**

: Describir el procedimiento de cobertura radicular con colgajo de reposición coronal (CRC) asociado a injerto de tejido conectivo (ITC) para el tratamiento de recesiones gingivales múltiples (RGM), con un abordaje frontal modificado en área estética.

**Reporte de caso:**

Paciente femenino que presenta RGM en región estética antero-superior. El abordaje elegido fue un CRC de abordaje frontal modificado más ITC. El ITCS se suturó en las papilas anatómicas desepitelizadas de las piezas con recesiones y el colgajo fue desplazado coronalmente y estabilizado mediante puntos de sutura suspensorios. Se efectuó el tratamiento para la herida palatina y se indicaron cuidados posoperatorios. El CRC asociado al ITC permitió resultados clínicamente predecibles y estables a largo plazo en RGM.

**Conclusión:**

La asociación del CRC de abordaje frontal modificado y el ITC parecen ser eficaces en el tratamiento de RGM en áreas estéticas, ya que logran cobertura radicular y estabilidad un año después de la cirugía.

## Abstract (en)

**Objective:**

To describe the root coverage procedure with a coronal advanced flap (CAF) associated with connective tissue graft (CTG) for the treatment of multiple gingival recessions (MGR) with a modified frontal approach in an aesthetic area.

**Case report:**

A female patient presents with MGR in the anterior-superior aesthetic region. The approach chosen was a modified frontal CAF plus CTG. The CTG was sutured to the de-epithelialized anatomical papillae of the teeth with recessions, and the flap was displaced coronally and stabilized with sling sutures. Treatment was performed for the palatal wound, and postoperative care was indicated. CAF associated with CTG allowed for clinically predictable and stable long-term results in MGR.

**Conclusion:**

The combination of modified frontal approach CAF and CTG appears to be effective in the treatment of MGR in aesthetic areas, achieving root coverage and stability one year after surgery.

## INTRODUCCIÓN

La recesión gingival (RG), definida como el desplazamiento apical del margen gingival con relación a la unión cemento-esmalte (UCE) y la consiguiente exposición de la superficie radicular ^1^, es un defecto mucogingival atribuido a diversas patologías, condiciones o factores que pueden ser localizados o generalizados ^2^. Todas las superficies dentales pueden experimentar esta pérdida de inserción clínica que constituye un problema funcional y estético; además, podrían estar asociadas a hipersensibilidad dental, lesiones cervicales no cariosas y retención de placa bacteriana ^2^^-^^4^. Adicionalmente, la literatura nos indica que la RG no tratada tiene un alto riesgo de progresión, incluso si se mantiene una adecuada higiene oral ^5^. 

La RG es un hallazgo clínico altamente prevalente en la población y se presenta mayormente en adultos, motivo por el cual muchos pacientes buscan con frecuencia tratamiento principalmente cuando se trata de la zona anterior maxilar ^6^^-^^8^. La cirugía plástica periodontal para la cobertura radicular de RG unitarias o múltiples ha experimentado un creciente avance que evidencia diferentes abordajes e inclusión de biomateriales ^2^^,^^5^^,^^9^. El objetivo de este tratamiento, además de lograr cobertura radicular, mejorar el fenotipo gingival y el ancho de la encía queratinizada ^9^, también se enfoca en el resultado estético final en zonas anteriores ^10^^,^^11^. Las recesiones gingivales múltiples (RGM) son defectos que presentan mayor complejidad quirúrgica debido a la amplitud del área a tratar y la variabilidad anatómica, al incluir piezas dentarias anteriores, posteriores o ambas, y sus respectivas características anatómicas adyacentes ^2^^,^^5^. Asimismo, se debe considerar el tamaño del injerto de tejido conectivo que se obtendrá de la mucosa del paladar ^12^^,^^13^. 

Los enfoques más utilizados para el tratamiento de las RGM incluyen al colgajo de avance coronal y la técnica de túnel. Sin embargo, el abordaje elegido debe individualizarse considerando el tamaño de cada RG, la disponibilidad de tejido queratinizado, el fenotipo periodontal, la disponibilidad de la papila interproximal, las variables anatómicas y las expectativas del paciente. Cuando las características de las recesiones gingivales son relativamente homogéneas, existe suficiente tejido queratinizado, se observan dimensiones favorables de la papila interproximal (RT1/Cairo o Miller I-II) y se busca alcanzar la máxima cobertura radicular ^14^^,^^15^, los estudios recomiendan el colgajo de reposición coronal (CRC) asociado a un injerto de tejido conectivo (ITC). Según la evidencia actual, este es considerado el tratamiento patrón oro, con resultados predecibles y estables en el tiempo ^9^^,^^16^^,^^17^. Existen diferentes técnicas y modificaciones para el tratamiento de las RGM ^2^^,^^5^^,^^9^, pero las dos más utilizadas son el abordaje lateral, que es la técnica que inicialmente se propuso y se concentra en tratar las RGM de piezas que se encuentran en un único cuadrante ^18^, y el abordaje frontal, que permite tratar piezas de diferentes cuadrantes, como los incisivos centrales, laterales y caninos, en un solo acto quirúrgico, lo que disminuye la morbilidad posoperatoria y tiene un impacto positivo en los resultados reportados por los pacientes ^15^. En ambas técnicas, los autores no indican la realización de incisiones liberantes para un desplazamiento coronal del colgajo. 

Por lo tanto, el presente reporte de caso describe y discute el tratamiento de un caso de RGM en un área estética con un abordaje frontal, en el cual se modifica la técnica original realizando incisiones liberantes.

### Declaración de consentimiento

La paciente dio y firmó su consentimiento informado para la publicación de este informe y las imágenes adjuntas. Este reporte de caso siguió la declaración CARE para la descripción de casos clínicos ^19^.

### Pretratamiento 

La paciente recibió terapia periodontal no quirúrgica que consistió en detartraje supragingival con un dispositivo ultrasónico y una profilaxis/pulido dental dos semanas antes de la intervención quirúrgica. Además, recibió instrucciones de higiene oral para el correcto mantenimiento de la higiene bucal en casa y para corregir cualquier hábito traumático de cepillado dental que pudiera estar relacionado con la etiología de la recesión. 

Una semana antes del procedimiento quirúrgico, se registraron los siguientes parámetros clínicos periodontales: altura de la recesión (AR), profundidad de sondaje (PS) y nivel de inserción clínica (NIC) (Tabla 1).

**Tabla 1:** Parámetros clínicos periodontales al inicio, a los 2 meses y al seguimiento de 12 meses

	Seguimiento	1.4	1.3	1.2	1.1	2.1	2.2	2.3
NIC	Inicio	7	6	7	6	4	5	5
	2 meses	2	2	3	2	2	3	2
	12 meses	2	2	2	2	2	2	2
AR	Inicio	5	5	5	4	2	3	2
	2 meses	0	0	1	1	0	0	0
	12 meses	0	0	0	0	0	0	0
PS	Inicio	2	1	2	2	2	2	3
	2 meses	2	2	2	1	2	3	2
	12 meses	2	2	2	2	2	2	2

NIC: nivel de inserción clínica; AR: profundidad de recesión; PS: profundidad de sondaje.

## DESCRIPCIÓN DEL CASO

Una mujer de 37 años, sin antecedentes sistémicos, no fumadora, se presenta a la consulta odontológica particular y refiere que presenta exposición radicular en la región anterosuperior. Su principal incomodidad era la estética de su sonrisa. También refirió el uso de cepillo dental de cerdas duras. El examen clínico evidenció la presencia de RGM que abarcaba desde el primer premolar derecho hasta el canino izquierdo, clasificadas como RT1 de Cairo ^3^ (Figura 1). Se analizaron los posibles abordajes quirúrgicos y se estableció como plan de tratamiento la técnica quirúrgica del colgajo de abordaje frontal con ITC. Sin embargo, en el examen clínico también se pudo observar que la pieza 2.4 presentaba una corona metal porcelana. Por tanto, si se realizaba un abordaje frontal, tal como lo describen Zucchelli y Sanctis ^15^, se tendría que incluir en el diseño del colgajo dicha pieza, que no presentaba una RG y tenía una corona, con lo que la predictibilidad de estabilidad del colgajo no sería la más adecuada. En consecuencia, dentro de la planificación del caso, se decidió realizar incisiones liberantes a distal de la pieza 2.3, a fin de no comprometer la pieza 2.4. La Figura 2 muestra un resumen de la planificación y el diseño del colgajo modificado propuesto para tratar este caso de RGM.

**Figura 1:**
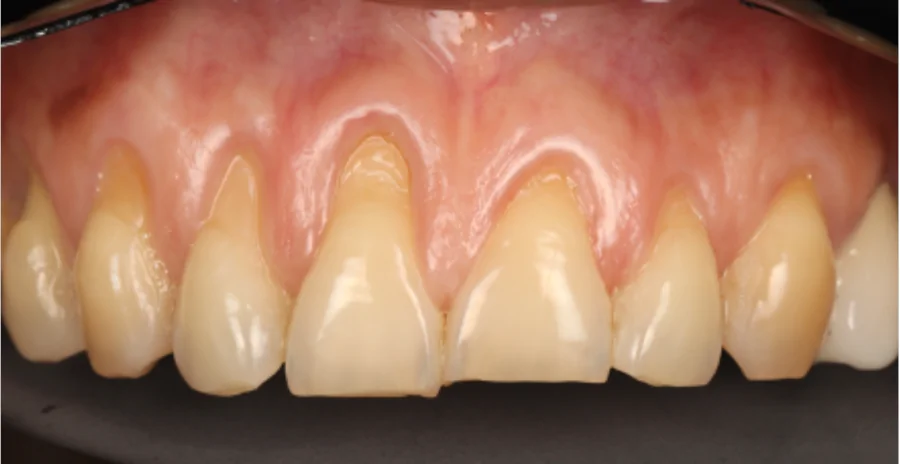
Recesiónes gingivales múltiples en el maxilar anterior.

**Figura 2:**
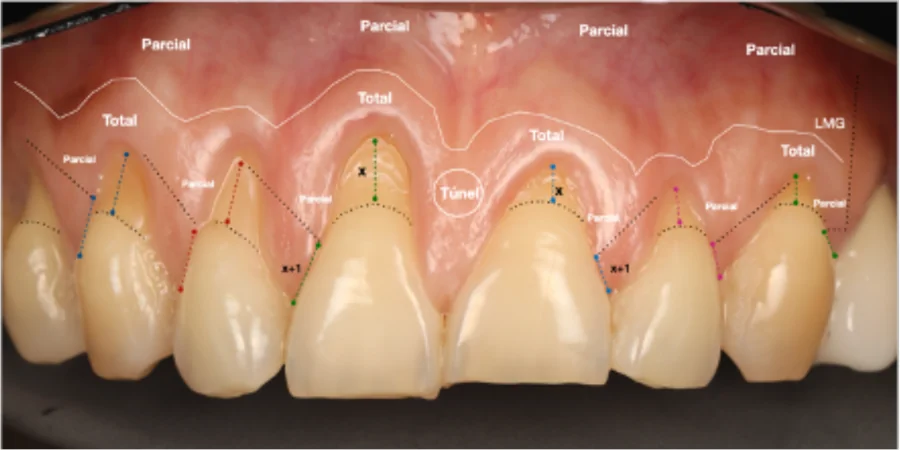
Diseño del colgajo mediante incisones oblicuas tomando como referencia los incisivos centrales, modificación del colgajo mediante incisión liberante a distal de la pieza 2.3 y distal de la pieza 1.4, y elevación del colgajo a espesor parcial en las papilas quirúgicas, total apical a la recesión hasta la línea muco gingival (LMG) y parcial en la mucosa alveolar.

### Procedimiento quirúrgico

La asepsia del paciente se realizó de manera intraoral con gluconato de clorhexidina al 0.12% y, de modo extraoral, con solución de yodo povidona. Luego, se colocó un campo quirúrgico fenestrado estéril. 

Se inició con la aplicación de la técnica de anestesia local infiltrativa con articaína al 4% con epinefrina 1:100000. Respecto del tamaño de las recesiones gingivales y la extensión del área a tratar, se determinaron las piezas dentarias guía para el abordaje de este caso, que fueron los incisivos centrales. De acuerdo con el tamaño de la RG de cada pieza dentaria, medida con una sonda periodontal, se sumó 1 mm para determinar el inicio de las incisiones oblicuas interdentales tomando como referencia el vértice de las papilas, y se repitió esta secuencia hacia distal (Figuras 2 y 3). Las incisiones oblicuas fueron unidas con incisiones sulculares a lo largo de los márgenes gingivales, todo esto realizado con hojas de bisturí número 15c (Swann Morton®). La técnica fue modificada al realizar dos incisiones verticales, una en distal de la pieza 2.3 y otra en distal de la pieza 1.4 (Figuras 2 y 3). 

**Figura 3:**
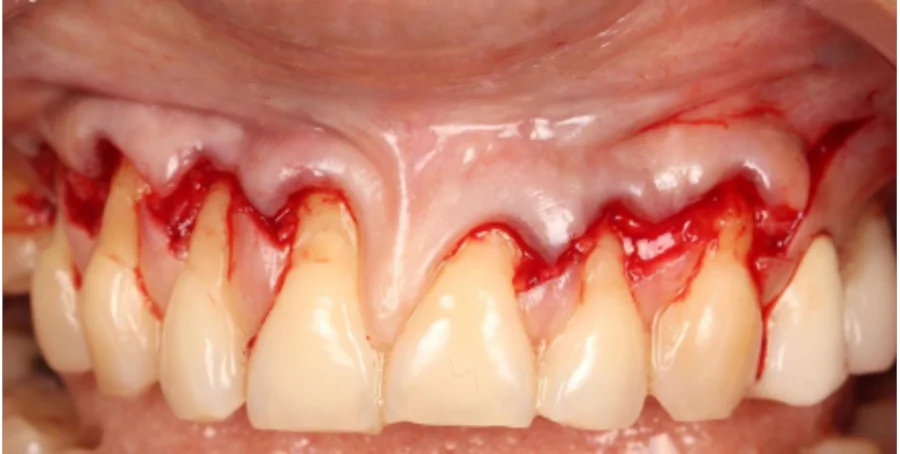
Diseño del colgajo mediante incisones oblicuas tomando como referencia los incisivos centrales, modificación del colgajo mediante incisión liberante a distal de la pieza 2.3 y distal de la pieza 1.4.

Se preparó un colgajo de espesor parcial-total-parcial de la siguiente manera: incisiones a espesor parcial desde las incisiones oblicuas para elevar las papilas quirúrgicas colocando la hoja de bisturí paralela al eje de cada diente; luego, a espesor total desde el margen apical hasta la línea mucogingival (LMG), con una cureta Molt (Hu-Friedy®), conservando el espesor vestibular y desprendiendo periostio que cubrirá la superficie radicular; y, finalmente, a espesor parcial en la mucosa alveolar para desinsertar las inserciones musculares. Estas incisiones fueron realizadas tanto en un plano profundo como uno superficial con el objetivo de conseguir un desplazamiento sin tensión del colgajo. La Figura 2 muestra un resumen del diseño modificado del colgajo y cómo fue su decolado parcial, total y parcial. 

La superficie radicular fue tratada de manera mecánica con curetas Gracey (Hu-Friedy®) solo en la superficie correspondiente a la pérdida de inserción clínica, para evitar daños en las fibras de tejido conectivo que aún están insertadas en el cemento radicular. Para culminar, entre los incisivos centrales, se aplicó la técnica de túnel empleando los tunelizadores (Hu-Friedy®) y, para proporcionar un correcto lecho quirúrgico a las papilas quirúrgicas, las papilas anatómicas fueron desepitelizadas con hoja de bisturí 15c (Swann Morton®) (Figura 4).

**Figura 4:**
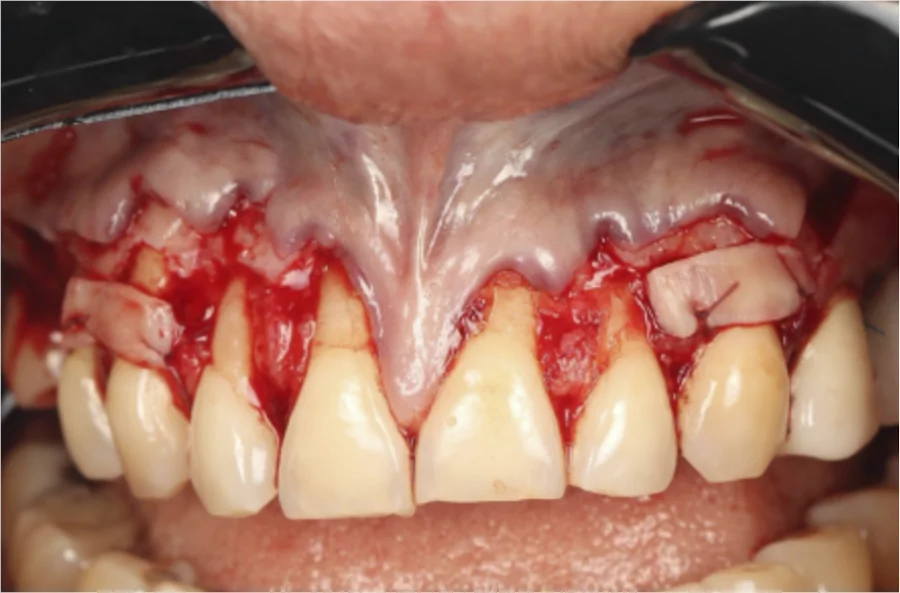
Colgajo decolado, papilas anatómicas desepitelizadas e injerto de tejido conectivo estabilizado a la altura de la unión cemento-esmalte con hilo de sutura reabsorbible 5/0 en las piezas 1.3 y 2.3

El ITC se extrajo de la mucosa palatina mediante la técnica de injerto gingival libre desepitelizado extraoralmente ^(2, 12)^. Con una sonda periodontal, se delimitó la zona de seguridad ^21^ a una distancia de 2 mm desde la unión cemento-esmalte, utilizando una hoja de bisturí número 15c (Swann Morton®). Se realizó el diseño rectangular conformado por dos incisiones verticales y dos incisiones horizontales, de acuerdo con el tamaño de la zona receptora, e incisiones a espesor parcial paralela al plano óseo para liberar por completo el injerto. Se realizó el tratamiento de la herida palatina con esponja de colágeno (HEMOTAMP®) y puntos de sutura en X para lograr hemostasia del área donante. El ITC se ubicó a la altura de la UCE y se estabilizó mediante puntos de sutura simples con hilo reabsorbible 5-0 (GLICOSORB®) en el periostio mesial y distal de las papilas anatómicas previamente desepitelizadas (figura 4). Finalmente, el colgajo fue desplazado coronalmente de manera pasiva cubriendo el ITC y las recesiones por completo, y se estabilizó mediante puntos de sutura suspensorios con hilo polipropileno 5-0 (TAGUM®) y puntos de sutura simples en las incisiones verticales (Figura 5).

**Figura 5:**
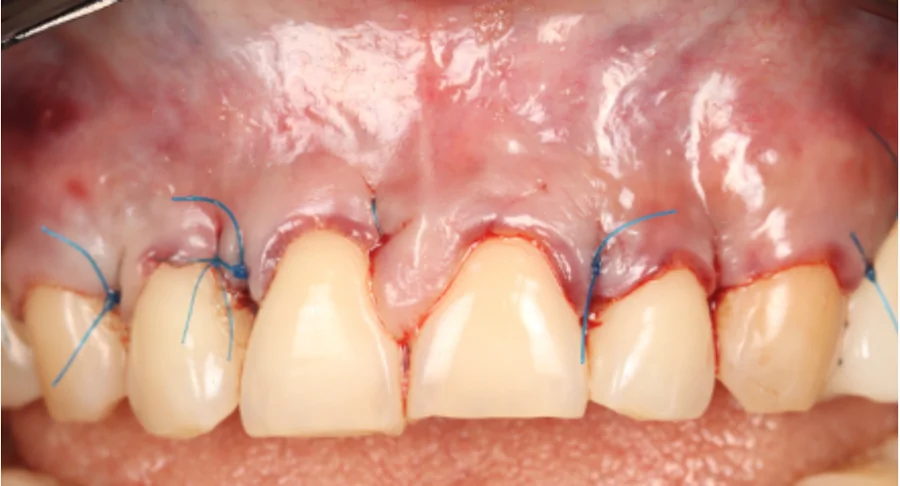
Puntos de sutura suspensorios con hilo de polipropileno 5-0 para un correcto desplazamiento del colgajo.

### Manejo posoperatorio

La paciente recibió terapia farmacológica. Para el cuidado posoperatorio se indicó no manipular la herida quirúrgica ni gesticular exageradamente, evitar el cepillado dental del área operada durante dos semanas, limitado esfuerzo físico y baja exposición al sol o calor. Se prescribió el uso de gluconato de clorhexidina al 0,12% dos veces al día durante 2 semanas. Los puntos de sutura del paladar se retiraron a los siete días y los puntos de sutura del colgajo se retiraron a los catorce días.

## RESULTADOS

El seguimiento clínico se realizó a los 2 meses (Figura 6) y un año después del procedimiento quirúrgico. Se observó una cobertura radicular completa en todas las piezas tratadas, con una adecuada cicatrización e integración del injerto (Figura 7). Los parámetros clínicos mostraron una reducción de la altura de la RG y ganancia del nivel inserción clínica (Tabla 1). Asimismo, la profundidad de sondaje se mantuvo estable sin evidencia clínica de bolsa periodontal.

**Figura 6:**
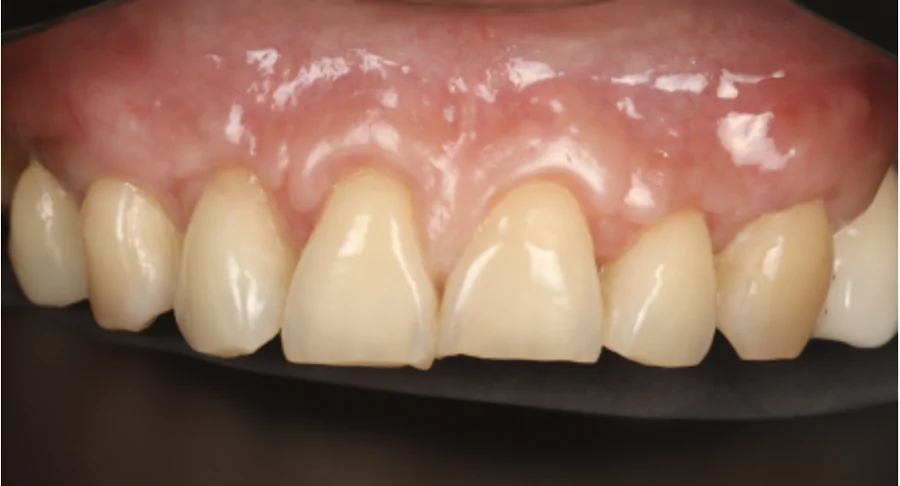
Dos meses después de la cirugía, cobertura radicular incompleta en las piezas 1.1 y 1.2

**Figura 7:**
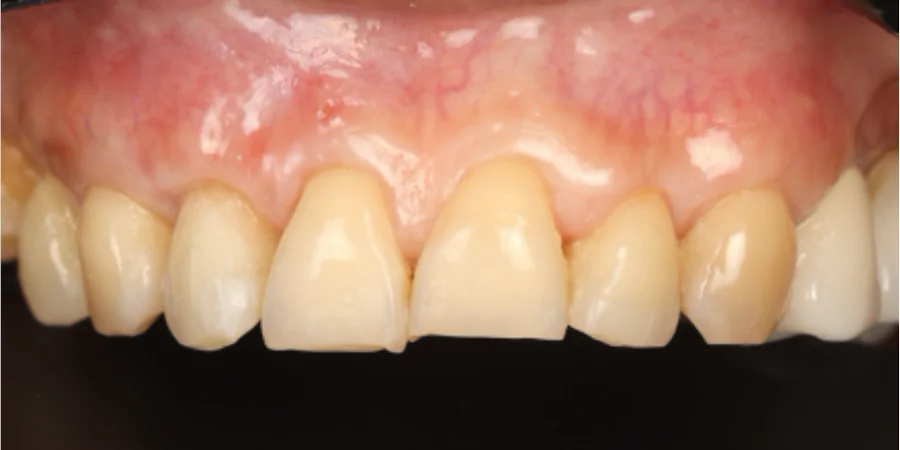
Cobertura radicular completa un año después de la cirugía.

## DISCUSIÓN

El tratamiento de la RG representa un área de la periodoncia ampliamente investigada debido a la alta prevalencia de esta afección en la población en general. En la revisión sistemática de Yadav *et al*., en el análisis por continentes, encontraron que Norteamérica presentó la mayor prevalencia (91,63%), seguida por Sudamérica (88.01%), con una prevalencia global del 84.92% para defectos de RG ≥1 mm ^7^. De manera similar, en el estudio de Marschner *et al*. se estimó una prevalencia global del 81.1% al aplicar el mismo punto de corte de RG ≥1 mm. Asimismo, para puntos de corte más elevados se reportaron prevalencias globales del 48,4% y el 16,2% para los puntos de corte de RG ≥3 mm y RG ≥5 mm, respectivamente. En los diferentes puntos de corte evaluados, Sudamérica registró tasas de prevalencia significativamente superiores en comparación con otras regiones ^8^. La salud periodontal representa un pilar fundamental de la salud bucal y cualquier alteración en la integridad de los tejidos puede ocasionar repercusiones importantes, e incluso irreversibles, que afectan la calidad de vida ^22^. 

La cobertura radicular de RGM representa un desafío clínico debido a la extensión del área quirúrgica, la variabilidad anatómica y el resultado estético esperado ^9^. Hofmänner *et al*., en su estudio, abordaron la predictibilidad de diversas técnicas quirúrgicas en el tratamiento de RGM, y determinaron que, en las clases I y II de Miller, el CRC y el CRC modificado produjeron cobertura radicular completa ^23^. Además, en las mismas clases, el CRC y el CRC modificado asociado a ITC produjeron una cobertura radicular completa más predecible y estable que con otros biomateriales ^23^. Tavelli *et al*. también informan resultados positivos en términos de cobertura radicular completa para el CRC en el tratamiento de RG unitarias o múltiples, y destacan mejores resultados para clases I y II de Miller en el maxilar superior y RGM ^17^. En el estudio de Chambrone *et al*. se mencionan como opciones válidas los diversos procedimientos de cobertura radicular, principalmente el CRC solo o acompañado de ITC u otro biomaterial; sin embargo, en casos donde además de cobertura radicular se busca ganancia en el ancho del tejido queratinizado la literatura recomienda el CRC sumado a ITC. Asimismo, se considera que el ITC promueve mayor estabilidad del margen gingival y posible inserción progresiva a largo plazo ^9^. 

Cairo *et al*. realizaron un ensayo para mostrar la eficacia clínica del CRC con o sin injerto de tejido conectivo, y obtuvieron un mejor resultado al usar ITC junto al CRC porque minimiza la contracción del margen gingival en dirección apical durante el posoperatorio. También observaron que el CRC sin ITC tuvo un mejor resultado estético en sitios con biotipo grueso, así como el CRC + ITC sigue siendo el estándar de oro para el tratamiento de RGM ^24^.

La obtención del ITC de la mucosa palatina puede generar desventajas como la cicatrización por segunda intención, la que se completa en un promedio de 2 a 4 semanas, tiempo en el cual el paciente puede experimentar limitaciones funcionales, incomodidad, hinchazón, dolor, ardor y limitaciones sociales, además de complicaciones inherentes al procedimiento ^11^^,^^25^. Por lo tanto, es crucial colocar ITC solo en las piezas dentarias que lo necesiten; por más que se trate de RGM, se debe evaluar cada pieza como unitaria. En el presente caso, si bien eran RGM, solo se utilizó ITC en las piezas 1.3 y 2.3. Es conocido por la literatura que se pueden obtener los mismos resultados clínicos en términos de cobertura radicular utilizando o no ITC cuando las piezas con RG presentan por lo menos 1 mm de altura y espesura de tejido queratinizado antes del tratamiento ^26^. Esto concuerda con nuestro reporte de caso, en el que después de un año de tratamiento se pudo observar recubrimiento radicular total en los dientes que recibieron y no recibieron ITC.

En el ensayo controlado aleatorizado de Zucchelli *et al*. se afirma que no hay diferencia estadísticamente significativa al realizar un CRC de abordaje lateral para el tratamiento de RGM con o sin incisiones de liberación vertical. Anteriormente se había informado que estas podrían limitar la nutrición del colgajo y generar cicatrices poco estéticas. Sin embargo, ambas técnicas ayudan a reducir la recesión y generan ganancia de inserción clínica. Cabe destacar que el CRC sin incisiones verticales tiene un mayor aumento de tejido queratinizado, un mejor resultado estético y menos complicaciones ^20^. En el presente reporte de caso se llevaron a cabo incisiones liberantes con el objetivo de no comprometer una pieza que tenía una restauración metal porcelana. Por tanto, no se podría hacer una comparación directa con el estudio de Zucchelli, ya que aquel compara la realización de incisiones liberantes o no con el colgajo de abordaje lateral, mientras que en este caso se utilizó el abordaje frontal. 

El estudio clínico de Zuccelli mostró diferencias estadísticamente significativas entre el grupo donde se realizó y no se realizó incisiones liberantes. De 16 pacientes tratados en el grupo donde se realizaron incisiones liberantes, 6 desarrollaron cicatriz (queloide) ^20^. Esa observación clínica no se encontró en ningún paciente al que no se le practicaron incisiones liberantes. En este reporte de caso, tras un año de seguimiento, no se observó ninguna cicatriz, lo cual puede deberse a que, si bien se llevaron a cabo incisiones liberantes, estas fueron superficiales y con la hoja del bisturí en una angulación de 45 grados.

Algo interesante para resaltar en este reporte de caso es que, a los dos meses de evaluación clínica, las piezas 1.1 y 1.2 aún presentaban 1 mm de RG. Sin embargo, después de un año de tratamiento, se pudo observar que ambas piezas ya no presentaban RG. Este fenómeno es descrito en la literatura como *creeping attachament*, el cual se define como la migración del margen gingival en dirección coronal sobre porciones de una raíz previamente expuesta, después de una cirugía mucogingival ^27^^,^^28^. Normalmente, se detecta entre 1 y 12 meses después de los procedimientos quirúrgicos, con una cobertura promedio de aproximadamente 1 mm ^29^. En este reporte de caso, las piezas que todavía presentaban RG dos meses después de la cirugía eran de 1 mm de altura. Es más probable que este fenómeno ocurra en pacientes jóvenes y cuando la recesión afecta a una sola raíz. Además, la posición del diente y la higiene bucal del paciente pueden ser factores que influyen en la migración coronal ^27^. Sin embargo, el mecanismo exacto y los eventos biológicos que conducen a la migración coronal aún se desconocen.

Aunque los hallazgos en este reporte de caso son prometedores, se necesitan estudios clínicos con mayor número de casos y seguimiento a largo plazo para confirmar la predictibilidad de este abordaje terapéutico.

## CONCLUSIÓN

Entre las limitaciones propias de un reporte de caso, el CRC de abordaje frontal modificado asociado a ITC colocado selectivamente mostró resultados clínicos y estéticos favorables en el tratamiento de recesiones gingivales múltiples Cairo RT1. Después de 12 meses de seguimiento, se observó cobertura radicular completa, estabilidad del margen gingival y mejoría de la inserción clínica en las piezas tratadas.

## Financiación:

Financiado por los propios autores.
